# Measured Dynamic Social Contact Patterns Explain the Spread of H1N1v Influenza

**DOI:** 10.1371/journal.pcbi.1002425

**Published:** 2012-03-08

**Authors:** Ken T. D. Eames, Natasha L. Tilston, Ellen Brooks-Pollock, W. John Edmunds

**Affiliations:** Department of Infectious Disease Epidemiology, London School of Hygiene and Tropical Medicine, London, United Kingdom; Pennsylvania State University, United States of America

## Abstract

Patterns of social mixing are key determinants of epidemic spread. Here we present the results of an internet-based social contact survey completed by a cohort of participants over 9,000 times between July 2009 and March 2010, during the 2009 H1N1v influenza epidemic. We quantify the changes in social contact patterns over time, finding that school children make 40% fewer contacts during holiday periods than during term time. We use these dynamically varying contact patterns to parameterise an age-structured model of influenza spread, capturing well the observed patterns of incidence; the changing contact patterns resulted in a fall of approximately 35% in the reproduction number of influenza during the holidays. This work illustrates the importance of including changing mixing patterns in epidemic models. We conclude that changes in contact patterns explain changes in disease incidence, and that the timing of school terms drove the 2009 H1N1v epidemic in the UK. Changes in social mixing patterns can be usefully measured through simple internet-based surveys.

## Author Summary

Changes in patterns of social mixing can result in changes in epidemic behaviour; this was observed during the 2009 influenza pandemic, in which the epidemic declined during school holidays and grew during term time. Until now, little information has been available to quantify how people's mixing patterns change over time. Here, we present the results of an internet-based survey of social mixing patterns that was carried out in the UK throughout the 2009 pandemic. We show that school holidays resulted in a substantial drop in the number of social contacts made each day, particularly between children. To test whether these measured patterns of social mixing could explain the observed epidemic, we used our mixing data in a simple mathematical model of influenza spread. We found that changing social contact behaviour could explain levels of infection in the community, and conclude that the timing of school terms was responsible for the shape of the influenza epidemic.

## Introduction

Seasonal changes in patterns of social contacts have a marked influence on the spread of infectious diseases. In particular, the patterns of school terms and holidays affect the incidence of infections with a significant impact on school-age children, including measles, pertussis, and influenza [1]–[6].

Mathematical models can be used to explain and attempt to predict the spread of infectious diseases; however until recently a lack of data about social contact patterns has restricted the applicability of these models. In 2008 results were published from the POLYMOD study, a social contact survey involving participants in 8 European countries [7]; this study described patterns of social mixing, quantifying the tendency of people to mix with others of a similar age, and showing that the highest levels of contact were between children. These data have been used to model close-contact infectious diseases, and have been found useful in explaining observed patterns of incidence [2], [5], [7]–[10].

Important factors are still missing from available datasets. One such factor is good information about how social contact behaviour varies over time. On an individual level, there is day-to-day variation in social behaviour [11], and incidence data suggest that there are population-level changes resulting from events such as school holidays [1]–[4], [6]. As part of the POLYMOD study, some data were collected during the school holidays, demonstrating significant changes in contact patterns during holiday periods [12]. Studies focusing on school-age children have confirmed that children make substantially fewer contacts on average during the holidays and at weekends than when at school [13]–[16]. However, there is a general lack of information about temporal changes in contact patterns, in particular quantifying the impact of school holidays on contact behaviour within the population as a whole. In the absence of these data, mathematical models of disease spread have been obliged to make a range of plausible assumptions about how to model the impact of school holidays [2]–[4], [6], [17]–[19]. Here, we present the results of a longitudinal population-level social mixing survey and use these data to parameterise an age-structured model of H1N1v incidence.

In April 2009, H1N1v influenza emerged in the Americas. Over the next few months, this virus spread around the globe, causing millions of cases worldwide. The UK experienced two distinct peaks in incidence, one in July 2009, and another in October 2009 [1], [2]. Serological data collected during the epidemic suggest that, in some parts of the UK, over 40% of children aged 5–14 were infected before the end of the first wave of infection [20], with an estimated cumulative incidence over the second epidemic wave in this group of 59% [21]; these serological data suggest that the great majority of cases were not captured in incidence estimates derived from clinical surveillance [1], even though such estimates may give a good indication of incidence trends.

The UK flusurvey (www.flusurvey.org.uk) was developed as an internet-based tool to augment existing influenza surveillance [22], [23], most of which depends on recording healthcare usage by symptomatic individuals [1], [24], [25] and so misses individuals with influenza-like-illness (ILI) who do not seek medical attention. The UK flusurvey is an attempt to record ILI incidence that does not depend on ill individuals seeking healthcare [23]. As well as estimating incidence trends [26], flusurvey data have been used to estimate the effectiveness of influenza vaccination [27]. Flusurvey participants were also asked about their social contact behaviour. Here we describe the results of the social contact survey carried out during the H1N1v epidemic in the UK. We describe changes in contact patterns that took place during the pandemic; in particular, we quantify the impact of school holiday periods. In order to test whether this internet-based social contact survey captures epidemiologically-relevant patterns of social interactions, we use the measured dynamic contact patterns in a simple mathematical model of influenza spread, and explore their ability to explain the observed patterns of incidence. We find that a relatively simple model, parameterised by our age-structured mixing data, gives a good match with observed patterns of incidence.

## Results

### Contact Survey

The contact survey was completed 9,261 times by 3,338 individuals, many completing it multiple times. 104 surveys were excluded from further analysis because of missing age information; the analysis that follows is based on the remaining 9,157 reports. The data can be found in the Supporting Information, Dataset S1. As expected, the majority of reports were completed by adults during the school term time (Table S1 in Text S1). We have therefore not further subdivided the school-aged groups or to attempted to distinguish between different holiday periods (e.g. summer holiday and autumn half term holiday).

**Figure 1:**
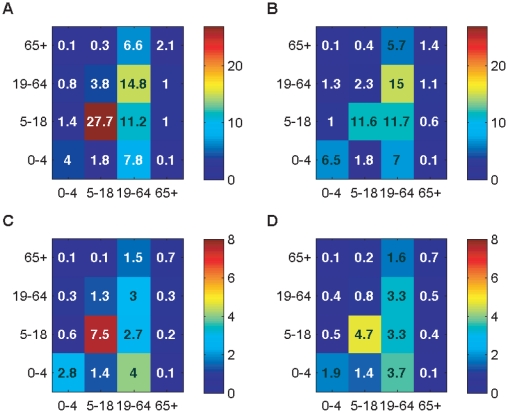
Social contact matrices. Values and colours show the mean number of contacts per day reported between each age group. In each panel, the participant's age group is shown on the vertical axis, that of their contacts on the horizontal axis. The four panels show patterns of A: conversational contacts during school term time; B: conversational contacts during school holidays; C: physical contacts during school term time; D: physical contacts during school holidays.

Fig. 1 shows the impact of school holidays on the social mixing patterns of the population. Both for conversational and for physical contacts the most obvious change was in the number of interactions between school-aged children. School holidays had a much smaller effect on the number of contacts made by or with other age groups.

There was a large, highly significant, reduction during the school holidays in the daily average number of conversational contacts made by those aged 5–18 (from 41.2 during term time to 24.8 during the holidays, p = 0.001; Table 1). Older age groups reported a small, but statistically significant, change. There were fewer physical contacts than conversational contacts reported, and the reduction in the number of physical contacts reported by school children during school holidays (from 11.0 to 8.9) was not statistically significant.

**Table 1:** Daily contact numbers.

	number of conversational contacts			number of physical contacts		
age group	school term time	school holidays	p	school term time	school holidays	p
0–4	13.8 (14.6)	15.4 (24.1)	0.701	8.2 (6.8)	7.1 (10.3)	0.750
	12 [5, 18]	7 [4, 17.5]		7 [3, 12]	3 [2, 9]	
5–18	41.2 (62.4)	24.8 (38.9)	0.001	11.0 (21.1)	8.9 (14.0)	0.342
	14 [6, 55]	9.5 [6, 26]		6 [3, 13]	5 [2, 9]	
19–64	20.5 (32.7)	19.6 (35.6)	0.017	5.0 (14.7)	5.0 (14.0)	0.633
	12 [6, 21]	12 [6, 21]		2 [1, 5]	3 [1, 5]	
65+	9.1 (19.6)	7.6 (8.3)	0.014	2.4 (3.3)	2.7 (4.2)	0.525
	4 [2, 9]	6 [3, 10]		1 [1, 3]	2 [1, 3]	

Summary of the number of daily contacts reported by participants in each age group, comparing term time with school holidays. For each age group, the mean (standard deviation), and median [inter-quartile range] are shown. p-values give the significance level for differences in number of contacts reported in school term time and school holidays.

### Model Performance

**Figure 2:**
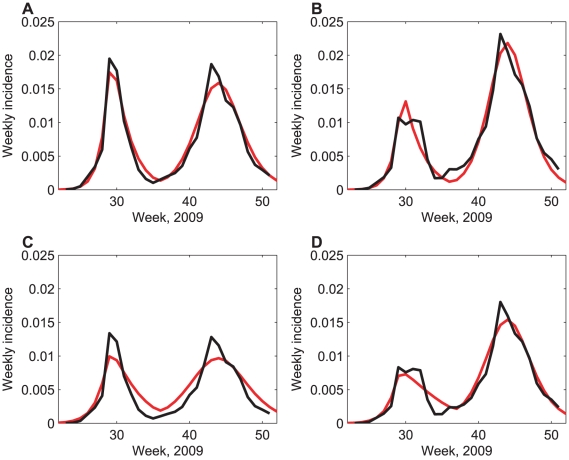
Incidence estimates, comparing models and data. A comparison of estimated per-capita weekly incidence data (black) and best-fitting model output (red). The four panels show A: model using patterns of conversational contacts fitted to HPA incidence estimates; B: model using patterns of conversational contacts fitted to flusurvey-adjusted incidence estimates; C: model using patterns of physical contacts fitted to HPA incidence estimates; D: model using patterns of physical contacts fitted to flusurvey-adjusted incidence estimates. Best-fitting parameter sets and fits using bootstrapped matrices can be found in Table S2 in Text S1, and Figs S1 and S2.

Models parameterised using these measured mixing patterns were fitted to estimated incidence curves (Fig. 2). While models parameterised using both conversational and physical contact patterns broadly capture observed incidence, the patterns of conversational contacts appear to provide a better fit to incidence data than patterns of physical contacts. In particular, models parameterised using physical contact patterns cannot capture the timing and depth of the trough in incidence at the end of the summer holidays. The model fits are similar whether using Health Protection Agency (HPA) or flusurvey-adjusted incidence estimates.

**Figure 3:**
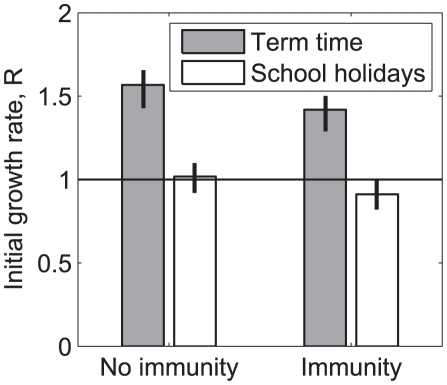
The impact of school holidays on epidemic growth rate. The impact of school holidays and prior immunity on initial epidemic growth rate predicted using the best-fitting model (using patterns of conversational contacts fitted to HPA incidence estimates) considering an epidemic that began during term time or during the school holidays, with and without measured levels of prior immunity. Comparable results from the other models can be found in Table S5 in Text S1. Lines show the range of model predictions using the low-difference and high-difference bootstrapped contact matrices.

An outbreak would have grown more slowly had it begun during the school holidays than during term time. During the holidays, in the absence of prior immunity, the initial growth rate of the epidemic, *R*, would have been approximately 35% lower than during term time (25% lower in the model using patterns of physical contact) – falling from 1.57 to 1.07. Prior immunity reduced initial growth rate by approximately 10%, to 1.42 in term time and 0.91 in the holidays (Fig. 3).

Estimated parameter values are reasonably consistent across the models used (Table S2 in Text S1), aside from the transmission rate per encounter, *τ*, which, as expected, is larger in the models using physical contact patterns. The value of the rescaling factor is estimated to be between 9 and 15.

**Figure 4:**
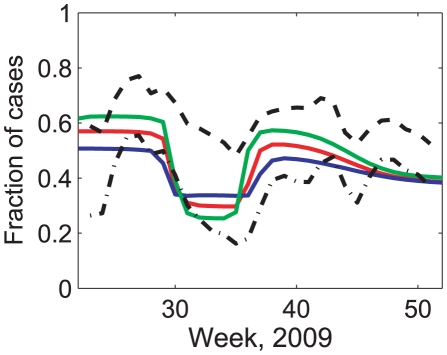
Incidence within younger age groups, over time. The fraction of incidence each week that occurs in younger people, as predicted using the best-fitting model (using patterns of conversational contacts fitted to HPA incidence estimates) and as reported in the HPA incidence estimate data. Incidence data showing the proportion of incidence in those aged under 25 (black, dashed) and under 15 (black, dash-dotted); model predicted fraction of incidence in those aged under 19 is shown in red; model predictions using the low-difference and high-difference bootstrapped contact matrices are shown in green and blue respectively.

The models suggest that around 30% of adults and over 50% of school-aged children had acquired immunity by the end of the outbreak (Table S3 in Text S1). Both models and incidence estimates indicate that incidence during school term time was dominated by those aged under 18, whereas during holidays the majority of cases were in adults (Fig. 4) [1]. The good agreement between the models and the data supports the usefulness of the mixing data obtained.

Mixing matrices generated from bootstrapping the original dataset suggest that the substantial change in contacts between term time and holidays is necessary for the model to be able to fit the incidence data, with low-difference bootstrap matrices resulting in models that fit the observed data less well (Figs S1, S2).

## Discussion

Substantial and significant changes in social contact patterns take place during school holidays. The greatest change is seen in school-aged children, who make approximately 40% fewer conversational contacts (95% CI 22–59%) each day during the school holidays than during term time.

These changes in social contact patterns have a large impact on the spread of infections. As the incidence patterns of the 2009 H1N1v epidemic in the UK show, incidence began to fall at the start of the holiday period and began to rise again when schools reopened. Models incorporating these dynamic contact patterns capture the observed dynamics of influenza, suggesting that the social contact patterns reported here are closely correlated to those relevant to the spread of influenza. The large fall in contacts during school holidays generates the observed decline in cases seen during the summer of 2009.

The models highlight the impact of prior immunity on epidemic behaviour, and suggest that, had the first cases arrived in the population during the school holidays, existing immunity in the population would have been sufficient to prevent the epidemic from taking off until schools reopened.

This work supports previous studies that suggest that school holidays are associated with significant changes in mixing patterns and in epidemic behaviour. The impact of holidays appears larger than some studies suggest [12], though not as large as others [13]–[16]. Different survey tools are likely to give different results: in contrast to surveys that use a detailed contact diary-based approach [7], [12]–[14], [28], [29] the method used here did not require participants to give additional details about each of the people they met, and thus there was no time-saving incentive towards recording fewer contacts; on the other hand, listing one by one all encounters may provide an aid to recall.

Several different methods of collecting social contact data have been used in other studies, including self-completed paper contact diaries [7], [11], [28], [29], network studies [8], [30], [31], electronic contact diaries [32], online contact diaries [29] and automated electronic proximity sensors [33]. All have been found to be useful, and none to be perfect. Perfect recall of all encounters is unlikely, especially for short-duration encounters [30]. Some studies have found electronic self-reported contact data to perform similarly to paper diaries [29], while others have found that more encounters are reported when using paper diaries [32]. In our study, we collected aggregate numbers of contacts (by age group and social setting), in order to reduce the time required to complete the surveys; a previous study suggests that this approach gives similar results to contact diaries if, as in our case, the recall period was short [28].

In common with other contact surveys [7], [11], data about the contact patterns of young children could be reported on their behalf by their parents, which may limit its reliability. Collecting contact data from young children is challenging though not impossible [13], [15], [31], and although our survey was designed to be straightforward to complete it was not possible to devise something that would be equally suitable for all age groups.

School closure has been suggested as an intervention to control infection, an idea that models have helped to explore [17]–[19]. Although this work demonstrates that scheduled school holidays have a large impact on transmission, school closure as a public health intervention may not have the same effect on social mixing patterns, since child care arrangements during unplanned, short-notice, closures may differ from those during school holidays. Unsurprisingly, there is only limited information available on this subject [15], [16], [34]. Furthermore, as was seen in the UK, it is likely that the epidemic would take off again once schools re-opened; thus school closure is more likely to be useful as a way to delay transmission than to prevent it altogether.

The models developed here suggest that a large fraction of the UK population was infected during the 2009 H1N1v epidemic. The same conclusion resulted from serological sampling that reported seropositivity by the end of the first wave of over 45% in children aged 5–14 in the regions of the UK first affected by the epidemic [20], and a cumulative incidence of 59% in this group over the second wave [21]. Interpretation of serological data is difficult, since not all those infected are expected to have seroconverted by the time of the sampling and blood samples used in these studies are not sampled at random [20], [21]. However, the models presented here, available serological data [20], [21], and other modelling work [2] all suggest that the original incidence figures dramatically underestimated the true number of infections. The models suggest that estimated influenza incidence only includes around 7–11% of all people infected. A number of factors may account for this, including mild or asymptomatic infections that would not have been diagnosed as ILI, imperfect test sensitivity, or poor estimates of the fraction of individuals with ILI who seek medical attention.

Ideally, there would be perfect incidence data to which to fit epidemic models. However, incidence estimates are not perfect, and serological surveys cannot give fine-scaled information about weekly incidence patterns. Here, we have used models appropriate to the level of incidence and behavioural data available and fitted models to incidence estimated in two different ways, in both cases drawing similar conclusions.

The social contact data used here are, likewise, imperfect. Participants in the flusurvey are not a random sample of the UK population, and we are unable to control for all biases in this self-selecting sample [23]. It would be interesting to be able to look at variations in contact patterns at a finer temporal resolution, such as comparing different holiday periods or detecting other temporal variations, but in this case the sample size, particularly of school age children, is not large enough to make this feasible. We cannot reasonably justify splitting up the most interesting and important groups – school-aged children – any further into, for example, primary and secondary school groups. It is planned to continue the UK flusurvey in future years, and it is hoped that wider recruitment will allow these issues to be explored more fully in due course.

We found that patterns of conversational encounters provided a better fit to incidence data than patterns of physical encounters. Some other studies have found that models using patterns of physical encounters provide a better fit to serological profiles [8], [9] though other studies do not find a difference between using physical and conversational encounter patterns [10]. Of course, fitting models to serological profiles that are the result of many years of potential exposure is not the same as fitting to short term incidence data. We found that the relatively small school-holiday change in numbers of physical encounters was unable to explain the sharp decline in incidence associated with the summer holiday period, an effect that may be less important when considering cumulative exposure over many years. Or it may simply be the case that conversational encounters provide a better proxy for interactions that led to the transmission of H1N1v than physical encounters.

The mathematical model of influenza transmission used here is extremely simple, with a population categorised into broad age groups roughly corresponding to normal patterns of work and school attendance. The model ignores geographical differences in transmission and incidence across the UK. The novel aspect of the model is that it makes use of measured changes in patterns of social contacts taking place between these groups as a result of the opening and closing of schools. The model is parameterised by data collected from an internet-based survey completed by a subset of the population of interest at the time of the epidemic.

Despite the caveats, the survey reported here is, to our knowledge, the only large-scale longitudinal study of population-level social contacts to have been carried out. We have shown that internet-based contact surveys can be used in large-scale studies. The fact that the contact data can be used in models to capture observed incidence patterns suggests that we have succeeded in quantifying epidemiologically relevant longitudinal social contact patterns.

## Methods

### Ethics Statement

Participation in this opt-in study was voluntary, and all analysis was carried out on anonymised data. The study was approved by the ethics committee of the London School of Hygiene and Tropical Medicine.

### The UK Flusurvey

The UK flusurvey was launched in July 2009, based on similar systems used elsewhere in Europe [22]. It ran from July 2009 until March 2010. Members of the public were encouraged to register via the flusurvey website and reported their symptoms (or lack of symptoms) each week. On registration, participants completed a background survey recording information about themselves including age, gender, and vaccination history. Participation in all parts of the flusurvey was entirely voluntary. Participants were prompted to continue to take part with a weekly email reminder. Further details about the flusurvey can be found in [23] and in Text S1.

Participants could also take part in a contact survey. This could be completed as often as participants chose; they were reminded of it each week, but its completion was not heavily advocated since the principal interest was in measuring incidence and behavioural response to infection [23], [26].

The contact survey was a simplified version of that used in other contact studies [7], [12]–[14], [16]: participants were asked two main questions: “How many people did you have conversational contact with yesterday?” and “How many people did you have physical contact with yesterday?” In each case, participants were asked to report the numbers of people they met in 4 different age groups (0–4; 5–18; 19–64; 65+), roughly corresponding to normal school and work attendance, and three different social settings (Home, Work/School/College, Other). Participants were asked to approximate larger numbers of contacts using in the following categories: 16–24; 25–49; 50–99; 100 or more; while we would have liked to collect precise numbers, it was decided that this would present an unrealistic recall challenge for participants. For larger numbers of contacts, in the analyses that follow the number of contacts was approximated by midpoint of these categories aside from the category “100 or more”, which was approximated by 150. Further details can be found in Text S1.

### Statistical Analyses

Participants were categorised into the same age groups as contacts (0–4; 5–18; 19–64; and 65+); time period was categorised as term time or school holidays.

To explore the influence of school holiday periods on the number and age distribution of contacts, accounting for multiple reports from participants who completed the contact survey multiple times, we used a population averaged negative binomial regression model with robust standard errors [35], [36]. Analyses were carried out separately for each age group of participants. Time period and gender were considered as explanatory variables, but gender was found not to be a significant factor and was subsequently omitted from the analyses. Analyses were carried out in Stata 11.

Because the weekly survey reminder email was sent to participants each Wednesday, and the contact survey asked about “yesterday's” contacts, most reports related to Tuesdays. Therefore, although a small number of surveys were completed on other days, day of the week was not included as a variable in the analysis.

### Dynamic Disease Model

A dynamic, differential-equation, age-structured, Susceptible-Exposed-Infectious-Recovered (SEIR) model [3] was used to investigate whether measured changes in contact patterns could explain the observed epidemic dynamics.

In this model, susceptible individuals become infected at a rate proportional to the number of contacts they have with infected individuals. Each contact (whether made during term time or holidays) has the same rate of transmission, *τ*; thus the rate at which a susceptible individual in age group *i* acquires infection is given by 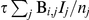, where *I_j_* is the number of infectious individuals in group *j*, *n_j_* the size of group *j*, and B*_i,j_* the number of contacts per unit time each individual in group *i* makes with individuals in group *j*.

When infected, an individual enters the exposed (latent) class, during which she is infected but not yet infectious. She then enters the infectious class at rate *ν*, then recovers at rate *g*. Because we consider events taking place over only a few months, ageing is not included.

The model is described by the following set of differential equations:
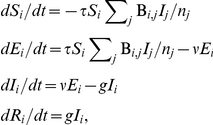
where *S_i_, E_i_, I_i_*, and *R_i_* are respectively the number of susceptible, exposed, infected, and recovered individuals in group *i*. The contact matrix {B*_i,j_*} is time dependent, representing differences in mixing patterns between school term times and holidays, taking values B^T^ *_i,j_* during term time and B^H^ *_i,j_* during the school summer holiday.

The initial growth rate of the epidemic, *R*, was calculated as the dominant eigenvalue of the next generation matrix *M*, with elements {M*_i,j_ = (τ/g)*B*_j,i_S_i_*/*n_i_*} (where, in the early stages of the epidemic *S_i_ = n_i_* in the absence of immunity) [3].

### Incidence and Immunity Data

The model was fitted to weekly incidence data based on individuals with ILI who sought medical attention [1]. Combined with laboratory testing of swabs taken from a subset of those who sought medical attention, these data are thought to give a good estimate of the number of cases of H1N1v with ILI who sought medical attention. To estimate the total number of H1N1v cases these observed cases must be scaled up to account for those individuals with influenza who do not seek medical attention.

We fit the model to two different estimates of weekly influenza incidence: one calculated by the HPA, using a scaling factor that was informed by flusurvey data made available to the HPA during the early part of the 2009 H1N1v pandemic [1]; the other using subsequent analysis of healthcare-seeking behaviour recorded by flusurvey users with ILI [27]. In reality, both estimates only provide approximations to true incidence trends. The advantage of the latter, flusurvey-adjusted, estimate is that it uses directly measured differences in healthcare-seeking behaviour between different age groups, and changes in this behaviour over time.

A large, unknown, number of people infected with influenza were either asymptomatic or displayed only mild symptoms, and as such would not have been recorded as ILI [2], [21], even if they had sought medical attention. In common with other modeling work, to account for this under-recording we apply a rescaling factor to the case estimates. Previous modeling work considered a rescaling factor of 7.5, 10, and 12.5, and concluded that a rescaling factor of 10 was reasonable [2]; here, we seek a more precise value for this parameter.

### Model Fitting

Two models were used: one using social contact pattern data relating to conversational encounters, and a second using data about physical encounters.

Weekly incidence as predicted by the model was fitted to estimated incidence data using a least-squares fit. Five model parameters were estimated: the transmission rate, the rescaling factor, the start of the epidemic and the beginning and end of the school holidays. Because of the rescaling factor included in the model, we fit to the shape of the incidence curve not its absolute value.

The best-fitting parameter sets (Table S2 in Text S1) were used to calculate the initial growth rate of the epidemic, *R*, for an outbreak beginning during term time and for one beginning during the school holidays, in the presence and in the absence of pre-existing immunity. Calculated values of *R* can be found in Table S5 in Text S1.

### Bootstrapping Contact Matrices

To explore the role of variability in the collected contact data, 1000 bootstrap copies of the dataset were generated, matching the original dataset in the number of responses from each age group in term time and holiday periods. These bootstrapped datasets were used to estimate a range of contact matrices describing term time and school holiday mixing patterns. It is not the absolute number of contacts but rather the change between holiday and term time contact patterns that is important for understanding the observed incidence; therefore, bootstrapped matrices were ranked according to the ratio of the term time and holiday epidemic growth rates. Models were fitted using those bootstrapped datasets that resulted in contact matrices that generated the 5^th^ and 95^th^ percentiles of this ratio (referred to as “low-difference bootstrap” and “high-difference bootstrap” respectively).

### Parameterisation

Serological testing in England indicated that a large number of people, particularly older people, had prior immunity to H1N1v [20]. In common with other interpretations [2], [20], we have assumed that a haemagluttination inhibition titre at or above 1∶32 provides immunity, and that the fraction of the population in each age group with levels greater than this before the epidemic is immune to further H1N1v infection. Values used in the models can be found in Table S4 in Text S1. To match the availability of serological data, the model population is parameterised to represent the population of England.

For simplicity, we use a latent period of one day and an infectious period of 1.8 days for H1N1v influenza in the UK, derived from previous modeling work by Baguelin et al [2]. Contact rates between age groups are taken directly from the flusurvey contact survey, and can be found in Text S1.

## Supporting Information

Figure S1 **Incidence estimates, comparing models and data.** Equivalent to Figure 2, using the low-difference bootstrap contact matrices. Comparison of estimated per-capita weekly incidence data (black) and best-fitting model output (red). The four panels show A: model using patterns of conversational contacts fitted to HPA incidence estimates; B: model using patterns of conversational contacts fitted to flusurvey-adjusted incidence estimates; C: model using patterns of physical contacts fitted to HPA incidence estimates; D: model using patterns of physical contacts fitted to flusurvey-adjusted incidence estimates. Best-fitting parameter sets can be found in Table S2 in Text S1, and values for contact matrices in Table S7 in Text S1.
(EPS)

Figure S2 **Incidence estimates, comparing models and data.** Equivalent to Figure 2, using the high-difference bootstrap contact matrices. Comparison of estimated per-capita weekly incidence data (black) and best-fitting model output (red). The four panels show A: model using patterns of conversational contacts fitted to HPA incidence estimates; B: model using patterns of conversational contacts fitted to flusurvey-adjusted incidence estimates; C: model using patterns of physical contacts fitted to HPA incidence estimates; D: model using patterns of physical contacts fitted to flusurvey-adjusted incidence estimates. Best-fitting parameter sets can be found in Table S2 in Text S1, and values for contact matrices in Table S7 in Text S1.
(EPS)

Figure S3 **Contact survey screen shot.** Screen shot from the contact survey, showing wording and layout of questions. Each entry in the matrix of encounter numbers consisted of a drop down menu. The number of physical encounters was asked similarly.
(TIFF)

Text S1 **The file Text S1 contains further information and parameters.** Section 1 contains the contact matrices (and bootstrapped contact matrices) as measured in the contact survey and as used in the dynamic disease model. Section 2 contains additional details about the survey design and participant recruitment.
(DOC)

Dataset S1 **The file DatasetS1.csv contains the data used in this manuscript.** Columns contain the number of reported conversational and physical encounters with each of the four age groups. The column “Term time” takes a value of 1 for surveys completed during school term time, and zero for surveys completed during the school holidays.
(CSV)
