# Supplementary figures and images for: Measured Dynamic Social Contact Patterns Explain the Spread of H1N1v Influenza
Source: PLoS Comput Biol. 2012 Mar 8;8(3):e1002425. doi: 10.1371/journal.pcbi.1002425 (PMC3297563; doi:10.1371/journal.pcbi.1002425)

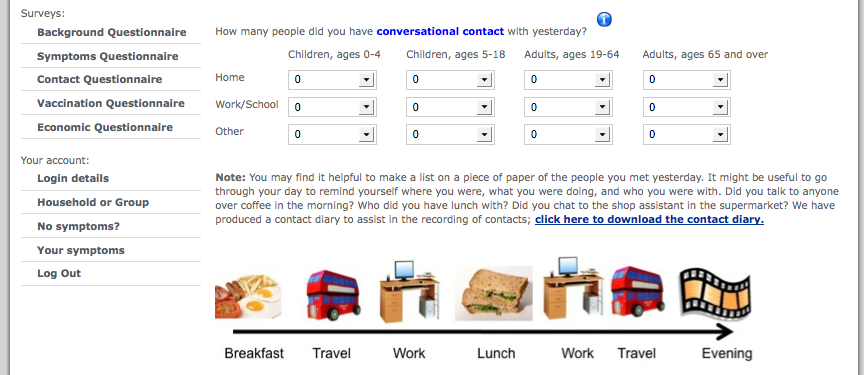

Supplement: Figure S3 — Contact survey screen shot. Screen shot from the contact survey, showing wording and layout of questions. Each entry in the matrix of encounter numbers consisted of a drop down menu. The number of physical encounters was asked similarly. (TIFF) [file pcbi.1002425.s003.tif]
