# Supplementary material for: Measured Dynamic Social Contact Patterns Explain the Spread of H1N1v Influenza
Source: PLoS Comput Biol. 2012 Mar 8;8(3):e1002425. doi: 10.1371/journal.pcbi.1002425 (PMC3297563; doi:10.1371/journal.pcbi.1002425)
Supplement: Text S1 — The file Text S1 contains further information and parameters. Section 1 contains the contact matrices (and bootstrapped contact matrices) as measured in the contact survey and as used in the dynamic disease model. Section 2 contains additional details about the survey design and participant recruitment. (DOC) [file pcbi.1002425.s004.doc]

Supporting Information for

**Measured Dynamic Social Contact Patterns Explain the Spread of H1N1v Influenza**

Ken T.D. Eames^1^*, Natasha L. Tilston^1^, Ellen Brooks-Pollock^1^, and W. John Edmunds^1^

London School of Hygiene and Tropical Medicine, Keppel Street, London, WC1E 7HT, UK.

* corresponding author; email Ken.Eames@lshtm.ac.uk

In this supporting information can be found the parameter values used in the models, including those taken from the literature, those fitted to incidence data, and the contact matrices calculated from the contact survey.

**1. Contact matrices**

The contact matrices generated from the dataset, as well as bootstrapped matrices used in the models, can be found below.

The contact survey recorded C*_i,j_*, the average number of people in age group *j* met each day by a person in age group *i*. In theory, each encounter is symmetric (i.e. each encounter between group *i* and group *j* should be recorded by someone in group *i* and by someone in group *j*), but in practice the sample is unlikely to be symmetric. To correct for differences in reporting by different groups, B*_i,j_* is calculated from the data as (*n_i_*C*_i,j_*+*n_j_*C*_j,i_*)/2*n_i_*. B^T^*_i,j_* and B^H^*_i,j_* are calculated separately using data collected during the school term and the school holidays respectively. Each 4x4 matrix describes the number of contacts per day reported within and between the 4 population age groups; in common with standard notation, age group of participant appears vertically (with the youngest at the top), that of the contact appears horizontally (with the youngest on the left); thus, for example, during term time each person aged over 65 reported on average 6.5673 conversational contacts per day with people aged between 19 and 64.

**1.1 Measured contact matrices**

**Conversational contacts, term time**

**Conversational contacts, school holidays**

**Physical contacts, term time**

**Physical contacts, school holidays**

**1.2 Bootstrapped contact matrices**

Bootstrapped contact matrices {B*_i,j_*}. For each contact type (conversational and physical), two pairs of matrices are shown; the low-difference set represents the 5^th^ percentile of the change in social mixing between term time and school holidays, as measured by the leading eigenvalue (proportional to the initial epidemic growth rate); the high-difference set represents the 95^th^ percentile.

**Conversational contacts, low difference**

**Conversational contacts, high difference**

**Physical contacts, low difference**

**Physical contacts, high difference**

**2. Survey and recruitment**

Flusurvey participants were recruited from the public through television, radio, and newspaper appearances. Those who registered to take part in the flusurvey were sent a weekly email newsletter, reminding them to report their symptoms (or lack of symptoms) each week. Registration was open to any interested member of the public. Participants were able to register on behalf of others, e.g. other family members.

The contact survey was an optional part of the flusurvey that could be completed as often as participants chose. The contact survey asked two questions about numbers of social contacts: “How many people did you have conversational contact with yesterday?” and “How many people did you have physical contact with yesterday?”. Conversational contact was defined as “talking face to face” and physical contact was defined as “skin to skin contact, e.g. a handshake, a kiss”.

For each of these questions, participants were asked to record the number of people they encountered in 4 different age groups and three different social settings (“Home”, “Work/School”, and “Other”). In each category, participants were asked to select the appropriate response from a drop-down list. See Fig. S3 for a screenshot of the survey.

To avoid demanding that participants who made large numbers of encounters recall each encounter, participants were asked to approximate larger numbers of encounters in each of the categories from the options: 16-24; 25-49; 50-99; 100 or more. Approximately 1% of contact number reports made use of one of these options, of which 17% were in the 50-99 or 100 or more categories. These approximated numbers of encounters were predominantly (71%) reported with people aged 19-64 in the Work/School or Other settings. These estimates were approximated by their midpoints, and the “100 or more” category by 150 for the analysis, although using other possibilities, e.g. approximating “100 or more” by 100, made no substantive difference to our conclusions.

Here, we present results from surveys completed between the launch of the flusurvey in July 2009 and its official end on 31^st^ March 2010.

The 9,157 contact surveys included in the analysis were completed by 3,249 distinct participants. 73% of participants completed only one contact survey, and 5.8% completed more than 10 contact surveys. The analysis presented in Table 1 accounts for clustering introduced by individuals who completed several contact surveys.

Table S1: number of reports included in the analysis, broken down by age group and time period.

| age group | term time | school holidays | total |
| --- | --- | --- | --- |
| 0-4 | 102 | 44 | 146 |
| 5-18 | 244 | 92 | 336 |
| 19-64 | 5535 | 2190 | 7725 |
| 65+ | 735 | 215 | 950 |
| Total | 6616 | 2541 | 9157 |

Table S2: best-fitting parameter values for each of the models used.

| interaction type, incidence dataset used | contact matrix used | *τ* | rescaling factor | start day | holiday start day | holiday end day | sum of squared difference |
| --- | --- | --- | --- | --- | --- | --- | --- |
| Conversational contacts, HPA estimates | average | 0.029 | 13.7 | 127 | 199 | 244 | 0.11 |
|  | low-difference bootstrap | 0.030 | 11.6 | 103 | 197 | 248 | 0.59 |
|  | high-difference bootstrap | 0.027 | 15.1 | 136 | 200 | 240 | 0.069 |
| Conversational contacts, flusurvey-adjusted estimates | average | 0.028 | 11.5 | 130 | 201 | 247 | 0.13 |
|  | low-difference bootstrap | 0.031 | 9.9 | 111 | 198 | 252 | 0.18 |
|  | high-difference bootstrap | 0.025 | 10.5 | 134 | 208 | 240 | 0.20 |
| Physical contacts, HPA estimates | average | 0.094 | 9.4 | 108 | 197 | 245 | 0.43 |
|  | low-difference bootstrap | 0.099 | 14.0 | 117 | 193 | 261 | 2.34 |
|  | high-difference bootstrap | 0.096 | 13.9 | 136 | 199 | 243 | 0.06 |
| Physical contacts, flusurvey-adjusted estimates | average | 0.096 | 9.0 | 119 | 197 | 252 | 0.14 |
|  | low-difference bootstrap | 0.099 | 9.2 | 117 | 194 | 262 | 0.84 |
|  | high-difference bootstrap | 0.088 | 9.6 | 130 | 207 | 241 | 0.16 |

Table S3: percentage of individuals no longer in the susceptible class.

| age group | conversational contacts, HPA estimates | | conversational contacts, flusurvey-adjusted estimates | | physical contacts, HPA estimates | | physical contacts, flusurvey-adjusted estimates | |
| --- | --- | --- | --- | --- | --- | --- | --- | --- |
|  | first wave | end | first wave | end | first wave | end | first wave | end |
| 0-4 | 8.8 [11.1 7.6 ] | 18.3 [18.8 18.1] | 6.9 [9.7 4.9] | 18.3 [19.0 15.4] | 9.9 [3.8 12.4] | 24.1 [27.6 30.7] | 9.0 [3.8 7.2] | 27.4 [27.4 25.7] |
| 5-18 | 23.8 [20.0 25.1] | 52.7 [44.1 56.9] | 19.9 [17.4 18.9 | 54.5 [47.3 53.3] | 21.2 [12.0 26.2] | 44.3 [41.0 56.3] | 19.3 [11.9 19.1] | 48.8 [40.7 51.3] |
| 19-64 | 18.3 [17.8 17.7] | 29.1 [28.0 28.0] | 16.5 [16.6 14.8] | 29.4 [29.3 25.1] | 15.8 [12.6 17.0] | 22.8 [22.6 26.1] | 15.3 [12.6 14.3] | 24.6 [22.5 23.3] |
| 65+ | 25.0 [24.7 24.9] | 28.1 [27.3 27.5] | 24.5 [24.4 24.1] | 28.2 [27.7 26.6] | 24.7 [23.7 24.6] | 26.7 [26.7 27.4] | 24.6 [23.7 23.9] | 27.3 [26.7 26.5] |

Percentage of individuals no longer in the susceptible class (either through infection or prior immunity), as predicted by the best-fitting parameters (predictions from the low-difference and high-difference bootstrap matrices shown in brackets). Number is given for the end of the first wave (when school reopened after the summer holiday) and the end of the epidemic.

Table S4: parameters used in the model

| parameter | value | source |
| --- | --- | --- |
| population size, 0-4 | 3.2 million | Census |
| population size, 5-18 | 9.9 million | Census |
| population size 19-64 | 32.3 million | Census |
| population size 65+ | 8.7 million | Census |
| initial proportion immune 0-4 | 0.0175 | [20] |
| initial proportion immune 5-18 | 0.0909 | [20] |
| initial proportion immune 19-64 | 0.1163 | [20] |
| initial proportion immune 65+ | 0.2327 | [20] |
| latent period | 1 day | [2] |
| infectious period | 1.8 days | [2] |
| initial proportion infectious | 0.000001 |  |

Table S5: model predicted epidemic growth rates.

| model | no prior immunity | | prior immunity | |
| --- | --- | --- | --- | --- |
|  | term time | holidays | term time | holidays |
| Conversational contacts, HPA estimates | 1.57 [1.43 1.66] | 1.07 [1.10 0.92] | 1.42 [1.29 1.50] | 0.91 [0.99 0.82] |
| Conversational contacts, flusurvey-adjusted estimates | 1.55 [1.44 1.54] | 1.01 [1.11 0.86] | 1.41 [1.30 1.40] | 0.90 [1.00 0.76] |
| Physical contacts, HPA estimates | 1.44 [1.32 1.64] | 1.07 [1.25 0.98] | 1.31 [1.20 1.49] | 0.97 [1.13 0.89] |
| Physical contacts, flusurvey-adjusted estimates | 1.47 [1.31 1.51] | 1.10 [1.24 0.90] | 1.33 [1.20 1.37] | 0.99 [1.13 0.82] |

Growth rates for best-fitting parameters using different models and different incidence estimates, considering a population with and without prior immunity. Predictions from the low-difference and high-difference bootstrap matrices are shown in brackets.
